# Auditory–Visual Matching of Conspecifics and Non-Conspecifics by Dogs and Human Infants

**DOI:** 10.3390/ani9010017

**Published:** 2019-01-07

**Authors:** Anna Gergely, Eszter Petró, Katalin Oláh, József Topál

**Affiliations:** 1 Institute of Cognitive Neuroscience and Psychology, Hungarian Academy of Sciences, 1117 Budapest, Hungary; petroeszti1989@gmail.com (E.P.); topaljozsef@gmail.com (J.T.); 2 Faculty of Education and Psychology, Eötvös Loránd University, 1064 Budapest, Hungary; olah_kata@yahoo.com

**Keywords:** cross-modal matching, dog, infant, intermodal cognition

## Abstract

**Simple Summary:**

Comparative investigations on infants’ and dogs’ social and communicative skills revealed striking similarity, which can be attributed to convergent evolutionary and domestication processes. Using a suitable experimental method that allows systematic and direct comparisons of dogs and humans is essential. In the current study, we used non-invasive eye-tracking technology in order to investigate looking behaviour of dogs and human infants in an auditory–visual matching task. We found a similar gazing pattern in the two species when they were presented with pictures and vocalisations of a dog and a female human, that is, both dogs and infants looked longer at the dog portrait during the dog’s bark, while matching human speech with the human face was less obvious. Our results suggested different mechanisms underlying this analogous behaviour and highlighted the importance of future investigations into cross-modal cognition in dogs and humans.

**Abstract:**

We tested whether dogs and 14–16-month-old infants are able to integrate intersensory information when presented with conspecific and heterospecific faces and vocalisations. The looking behaviour of dogs and infants was recorded with a non-invasive eye-tracking technique while they were concurrently presented with a dog and a female human portrait accompanied with acoustic stimuli of female human speech and a dog’s bark. Dogs showed evidence of both con- and heterospecific intermodal matching, while infants’ looking preferences indicated effective auditory–visual matching only when presented with the audio and visual stimuli of the non-conspecifics. The results of the present study provided further evidence that domestic dogs and human infants have similar socio-cognitive skills and highlighted the importance of comparative examinations on intermodal perception.

## 1. Introduction

Integration of information coming from several sensory modalities is essential for communication and individual recognition in several species. Because humans communicate mostly through auditory and visual channels, the intermodal relations between faces and voices are crucial for the development of linguistic, social, and emotional skills [1]. The so-called intermodal looking preference technique [2] is commonly used for studying intermodal cognition in humans [1] and non-human animals [3]. In these experiments, subjects are concurrently presented with two visual displays accompanied with a single auditory stimulus corresponding to one of them. Based on spontaneous preferences for looking at a specific visual stimulus, researchers can infer how participants match the pictures with the sound played. Using this paradigm, Walker-Andrews and co-workers [4] showed that six-, but not three-, month-old infants match unfamiliar human faces and voices based on gender information. It has also been shown that five- to seven-month-olds prefer to watch happy, sad, neutral, or angry facial expressions when a corresponding sound is played [5,6], and match faces and voices based on the age of the speaker [7].

In past decades, there has been a growing interest in studying infants’ cross-species intersensory perceptual and discriminative abilities. In their study, Lewkowicz and Ghazanfar [8] showed that four- and six-month-old infants are capable of intersensory matching of dynamic visual displays of unfamiliar macaque faces and calls, while 8- and 10-months-olds are not. The authors concluded that the developmental mechanism, which is initially broad enough to also perceive intersensory relations in cross-species events, is highly adaptive because it enables infants to recognise that particular faces and voices belong together. However, this initially broad face-voice matching ability starts to narrow after the first year of life, as infants gain more perceptual experience with human faces and voices, that is, the function of this mechanism becomes more specialised to features that are relevant in the infant’s environment. This so-called perceptual narrowing can account for older infants’ failure in the task described above [8]. Another study of intermodal relations across species investigated whether 6- to 24-month-old infants could match aggressive and nonaggressive canine barks with appropriate canine facial expressions [9]. In order to rule out the possibility of using temporal synchrony between the given bark and the mouth closing, infants were presented with static pictures of canine faces. The analysis of the proportion of total looking time data showed intermodal matching of barks and facial expressions in six-month-old infants. In contrast, 18- and 24-month-olds looked longer at the incongruent picture during the second half of the given trial, while 12-month-old infants did not show any looking preference toward either of the pictures. The analysis of the direction of infants’ first looks, however, indicated signs of intermodal matching by older (12-, 18-, and 24-month-old) infants, but not by 6-month-old infants. These results suggest that cross-species intersensory perception may not decline over time and highlight the importance of investigating preferential looking in such tests [9].

In order to understand the phylogenetic aspects of intermodal cognition, several studies examined primate species, offering detailed reports regarding the perception and integration of cross-modal information, such as the works of [10,11,12,13]. One study demonstrated spontaneous auditory–visual intermodal recognition of conspecifics, but not of heterospecifics in a chimpanzee (*Pan troglodytes*) [14]. Further investigations showed that chimpanzees performed equally well in recognising and matching the faces and voices of familiar adult conspecifics as those of humans [15]. In a comparative study, however, both chimpanzees and humans performed better in recognizing familiar conspecifics than familiar non-conspecifics [16]. It has also been reported that Japanese macaques (*Macaca fuscata*), rhesus macaques (*Macaca mulatta*), and squirrel monkeys (*Saimiri sciureus*) show some evidence of acoustic–visual intermodal matching of familiar con- and heterospecific individuals [17,18,19].

Some recent studies have focused on con- and heterospecific intermodal matching in domesticated animals, such as the works of [3,20,21]. Such investigations are fuelled by the idea that for domestic species, it is especially important to integrate multiple cues in order to form representations not just about their own species, but also about a morphologically very different species, humans. Using the violation of expectation paradigm, Proops and co-workers [22] showed that domestic horses seem to possess cross-modal representations of known conspecific individuals containing unique auditory and visual/olfactory information, and they are also capable of matching the face with voice of familiar people (handler) [20]. Similarly, dogs are also able to pair their owner’s portrait with their owner’s voice [21].

In the last 10 years, there has been a growing interest in dogs’ (*Canis familiaris*) cross-modal cognitive abilities, for example [23,24]. Using the intermodal looking preference paradigm, Faragó and colleagues [3] showed two dog pictures to adult pet dogs while a dog growl was played. Dogs looked sooner and longer at the picture showing a dog matched in size to the growling dog, which suggested that they might be able to form mental representations about the signaller with respect to its size. It has also been shown that dogs can spontaneously categorise potential human partners as male or female in a cross-modal (auditory–visual) preferential looking task. However, the accuracy of this voice-based categorisation was strongly affected by earlier social experience with humans [24].

Close co-existence, sharing the same living habitat, and analogies in canine and human socio-cognitive abilities make the dog a unique model for comparative cognition studies (see [25] for a review). It is widely accepted that domestication has equipped dogs with sophisticated infant-like sensitivity to respond to human visual communicative cues, including gaze direction and pointing gestures [26]. However, dogs’ ability to integrate audiovisual information in con- and heterospecific face processing is a still unexplored field of eye tracking research. Even more importantly, it is also unclear whether the observed similarities between dogs’ and human children’s social-communication skills hold true in respect to their cross-modal (voice–face) matching abilities. Despite the fact that numerous studies have investigated infants’ and dogs’ multimodal perception, such as the works of [1,3,9,27], none of them utilised a comparative method, which would allow direct comparison of cross-species intermodal cognitive abilities of dogs and humans. Such comparisons could test the evolutionary account for the emergence of cross-modal perception and could have the potential to provide new insights into dog–human interaction.

In the present study, we investigated whether adult dogs and 14–16-month-old human infants show auditory–visual matching of con- and heterospecific faces and voices. We employed a non-invasive eye tracking method that has been used successfully for investigating visual–spatial attention in dogs, such as in the works of [28,29,30,31,32], and infants (for a review, see [33]), but has not yet been applied to the study of cross-species intersensory perception in dogs and infants. Subjects in the present study were concurrently presented with a dog and a female human portrait, while auditory stimuli of female human speech and a dog’s bark were played back consecutively. Based on previous findings, we predicted that both dogs and infants would look first at the congruent picture (i.e., at the dog picture during the bark and at the human picture during the speech). However, only dogs were expected to gaze longer at the congruent picture, while infants were predicted to show a looking preference for the incongruent one [9]. We also expected that dogs would spend more time looking at the picture of a conspecific than looking at the human portrait [29,34].

## 2. Materials and Method

### 2.1. Ethical Note

This research was approved by the Human Research Ethics Committee (EPKEB) at Hungarian Academy of Sciences (No. 2015/23). In accordance with ethics approval, all parents and dog owners completed an informed written consent to participate in the study and all methods were performed in accordance with the relevant guidelines and regulations of the EPKEB and the current laws of Hungary.

### 2.2. Subjects

Twenty human infants (12 boys; 8 girls; mean age ± SD, 14.5 ± 1.2 months) from a database at the Institute of Cognitive Neuroscience and Psychology, Hungarian Academy of Sciences, and 42 adult pet dogs from 16 different breeds (19 males; 23 females; mean age ± SD, 3.4 ± 2.6 years) participated in the experiment. The dogs were recruited on a voluntary basis from the Family Dog Research Database. We obtained valid data for the analysis from 18 infants (11 girls; 7 boys; mean age ± SD, 14.37 ± 1.1 months) and 27 dogs (12 males; 15 females; mean age ± SD, 3.2 ± 2.1 years) (for the criteria of validity, see Data Analysis section). Parents of the infants also filled out a survey that included questions about their infants’ experience with dogs. There were only 2 infants (11.1%) from dog-owning families, and 6 infants (33.3%) had interacted with dogs only one to two times over the course of their lifetime, while the remaining 10 infants had no personal experience with dogs. Owners and parents of all participants gave informed consent and were instructed in advance how to behave and what to do during the test; however, they were unaware of the hypothesis of the study.

### 2.3. Apparatus

We collected eye gaze data using a Tobii X50 Eye Tracker (Stockholm, Sweden). The eye tracker had a constant 50 Hz sampling rate with 0.5–0.7 degree accuracy and 30 × 16 × 20 cm freedom of head movement. The stimuli were presented on a 17-inch LCD monitor positioned behind the eye tracker. The owner made the dog stand, sit, or lie down in order to get optimal eye-gaze data (at a distance of approximately 60 cm). The owner sat behind the dog and turned his/her head down while looking down and avoiding verbal interactions.

The parent was asked to sit down on a chair facing the apparatus (at a distance of approximately 60 cm) and to hold the infant on his/her lap. The parent was also instructed to close his or her eyes during the entire test procedure.

### 2.4. Stimuli

The visual events consisted of a pair of portraits of a domestic dog (Mudi, Photo Credit: Péter Pongrácz) and a caucasian female human downloaded from the Radboud Faces Database [35]. The auditory stimuli consisted of a dog’s bark and female human speech. The bark from a Hungarian herding dog breed (Mudi) was recorded by Pongrácz et al. [36] in the ‘Dog Alone’ situation (for details, see [36]), while the female human speech stimulus was obtained from a study of Zainkó and colleagues [37]. The human speech contained one semantically neutral sentence in Hungarian presented in a fearful manner in order to make the emotional content similar to the dog bark.

### 2.5. Procedure

#### 2.5.1. Calibration

The eye gaze recording was preceded by a five-point calibration phase following the infant calibration protocol of Clearview 2.5.1. During calibration, the dogs could see a video presentation in which a tweeting toy object appears, disappears, and reappears five times on different parts of the screen [28], while infants were presented with a moving, pulsing blue circle appearing five times on the same parts of the screen as the toy object for dogs. Only those subjects who reached the criteria for sufficient calibration (both eyes were captured by the eye tracker at least four out of the five calibration events) participated in the test trial.

#### 2.5.2. Test Trial

**Figure 1:**
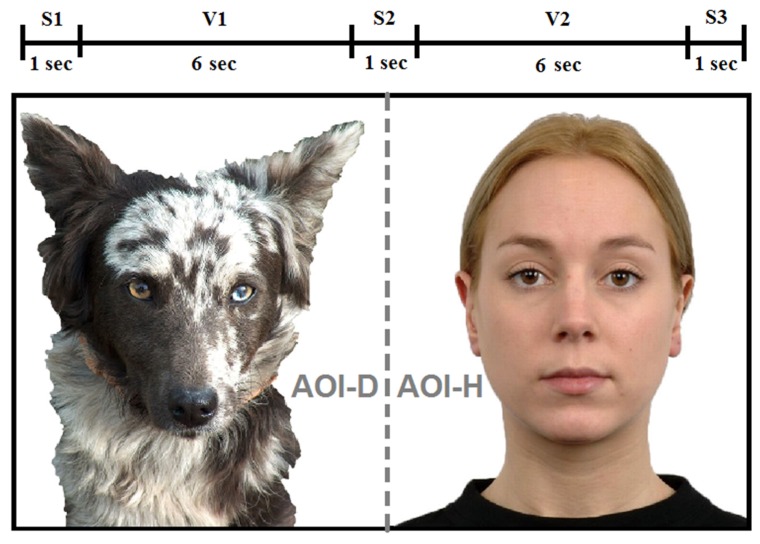
Experimental stimuli. S1, S2, S3 = silence; V1, V2 = vocalisation (i.e., dog bark/human speech); grey line shows the separation of the two areas of interest (areas of interest (AOI); dog = AOI-D, human = AOI-H).

The test trial started with a beeping cartoon animation that directed the participants’ attention toward the centre of the screen (4 s long; see [28]). Then, subjects were presented with a 17 s long test stimulus, which consisted of the following five phases (S1–3 and V1–2):

Simultaneous presentation of a dog and a female human portrait without adding sound (duration: 1 s, S1), then the dog bark (7 s) and female human speech (7 s) were played (V1 & V2) with a 1 s long mute break in between (S2). Finally, the dog and the female human images were presented for an additional second in silence (S3). The position of the portraits and the order of the replayed sounds were counterbalanced across subjects (Figure 1).

### 2.6. Data Analysis

Our statistical analysis was based on the eye gaze collected from two regions that were defined as areas of interest (AOI) during phase V1 and V2 (AOI-D = dog picture, AOI-H = human picture; see Figure 1). During the dog bark, AOI-D was considered voice congruent and AOI-H voice incongruent. During the human speech, AOI-H was considered voice congruent and AOI-D voice incongruent. The test trial was accepted as valid for analysis only if provided at least 80 ms eye gaze data from the screen (AOI-D + AOI-H) [29,30]. We were also interested in the direction of the first gazes of our participants upon hearing the auditory stimuli. For infants, following standard procedures, we focused on the direction of the first fixation (‘target area of first fixation’ variable), for which a 100 ms threshold was set. Because of the fixation time and gaze-pattern differences between dogs and infants [32,38], we analysed the direction of the first look that reached at least 20 ms in length in dogs (‘target area of first look’ variable). Taking these considerations into account, 27 dogs and 18 infants provided valid data and were included in the statistical analysis of the looking duration variable. All 18 infants provided valid data in both V1 and V2, while only 25 dogs provided valid looking data during phase V1 and V2. According to the criteria set for the target area of first look/first fixation variables, 17 infants and 23 dogs provided valid data in V1 and 17 infants and 23 dogs provided valid data in V2.

Raw looking data is available in S1 data.

Subjects’ looking behaviour was tested along two variables:

(i) Looking duration (ms): summary of looking durations in AOI-D as well as in AOI-H. Looking durations were calculated separately in V1 and V2 phases.

(ii) Target area of first look/fixation (0/1): because dogs’ and infants’ fixation times differ significantly [32,38], we decided to use different thresholds for the first look/fixation variable. For dogs, valid (min. 20 ms long) gaze data were first recorded separately at AOI-D or AOI-H in V1 and V2 phases. For infants, valid (min. 100 ms long) fixation data were first recorded separately at AOI-D or AOI-H in V1 and V2 phases. We scored each phase as 1 if the subject’s first look/fixation was recorded in AOI-D, and 0 if it was recorded in AOI-H.

To control for repeated measures, we applied random intercept generalised linear mixed-effect models (GLMMs) to the data using IBM SPSS 21, with subject ID included as a random grouping factor. Looking durations of the two species were analysed in separate models because of the difference in the fixation times and looking duration in dogs and humans [32].

For the target area of first look/fixation variable, the fixed explanatory variables included Vocalisation (Bark, Speech), Phase (V1, V2), Species (Dog, Infant), Vocalisation × Phase, Species × Phase, and Vocalisation × Species interactions. For the looking duration variable, the fixed explanatory variables included Vocalisation (Bark, Speech), AOI (Dog, Human), Phase (V1, V2), Vocalisation x AOI, AOI × Phase, and Vocalisation × Phase interactions.

The looking duration (ms) variable was analysed by GLMM with Gaussian error distribution and the first look/fixation (binary) variable was analysed by GLMM with binomial distribution. The binary model was not over-dispersed. All tests were two-tailed and the α value was set at 0.05. Sequential Sidak correction was applied in all post-hoc comparisons. Non-significant interactions and main effects were removed from the model in a stepwise manner (backward elimination technique).

## 3. Results

### 3.1. Target Area of First Look/Fixation

**Figure 2:**
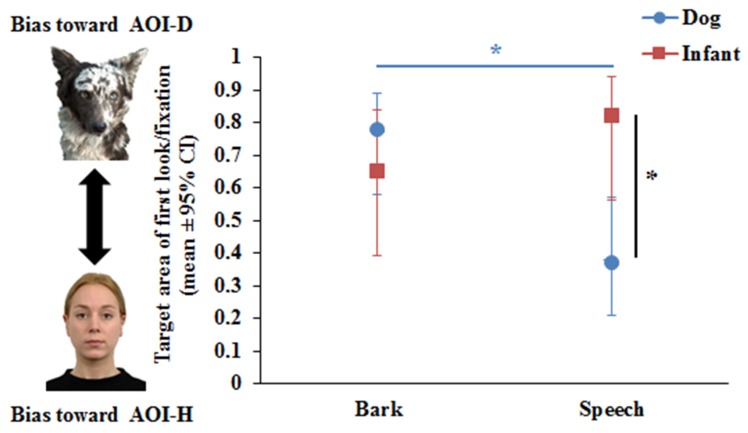
Dogs’ and human infants’ visual preferences as measured by first look/fixation at dog or human images during dog bark or human speech. * *p* < 0.05.

The Binary GLMM showed that Phase did not have a significant effect on the first look/fixation variable, either as a main effect (F_1,83_ = 0.29, *p* = 0.59) or in interaction with Species (F_1,74_ = 0.03, *p* = 0.85) and Vocalisation (F_1,74_ = 0.5, *p* = 0.48). The results of the final Binary GLMM showed a significant Vocalisation x Species interaction (F_1,77_ = 4.42, *p* = 0.039). Pairwise comparisons revealed that dogs (*p* = 0.001), but not infants (*p* = 0.24), showed auditory–visual matching. While dogs looked first at AOI-D during Bark and at the AOI-H during Speech (Figure 2), a different pattern was found for infants. Infants were more willing to fixate first at the voice incongruent AOI (AOI-D) (*p* = 0.001), while no such difference was found during Bark (*p* = 0.36) (see Figure 2).

### 3.2. Looking Duration

**Figure 3:**
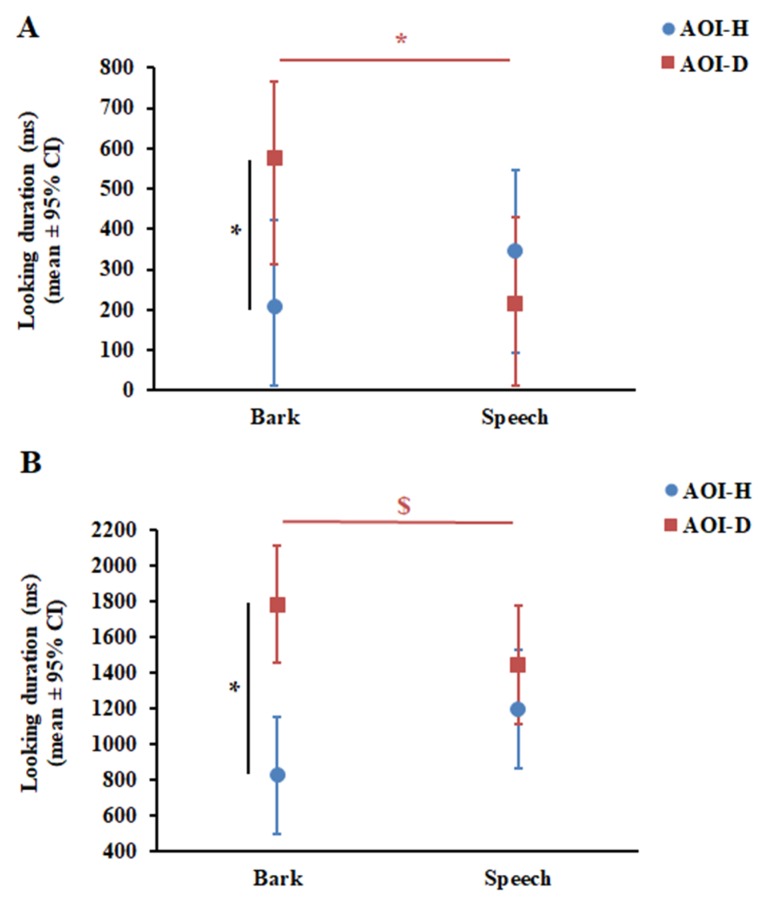
Results of looking duration in dogs (**A**) and infants (**B**). * *p* < 0.05. $ *p* = 0.09. CI—confidence interval.

The GLMM showed that Phase did not have a significant effect on dogs’ looking duration, either as a main effect (F_1,95_ = 0.54, *p* = 0.47) or in interaction with AOI (F_1,93_ = 2.01, *p* = 0.16) and Vocalisation (F_1,93_ = 1.04, *p* = 0.31). At the same time, the results of the final GLMM showed a significant Vocalisation × AOI interaction (F_1,96_ = 4.83, *p* = 0.03). Post-hoc pairwise comparisons revealed that dogs looked longer at AOI-D during Bark than during Speech (*p* = 0.028); however, they looked equally long at AOI-H during Bark and Speech (*p* = 0.39) (Figure 3A). In line with this result, dogs’ looking duration at AOI-D and AOI-H differed only during Bark (*p* = 0.023), but not during Speech (*p* = 0.43).

GLMM also showed that Phase did not have a significant effect on infants’ looking duration, either as a main effect (F_1,67_ = 2.07, *p* = 0.15) or in interaction with AOI (F_1,101_ = 0.11, *p* = 0.74) and Vocalisation (F_1,65_ = 0.22, *p* = 0.64). Similar to in dogs, the final GLMM showed a significant Vocalisation × AOI interaction (F_1,68_ = 5.28, *p* = 0.025) in infants. Pairwise comparisons revealed that infants showed only a tendency to look longer at AOI-D during Bark compared with Speech (*p* = 0.09) and, similar to dogs, they looked equally long at AOI-H during Bark and Speech (*p* = 0.13) (Figure 3B). Interestingly, infants looked longer at AOI-D compared with AOI-H during Bark (*p* < 0.001), while no such preferential looking was found during Speech (*p* = 0.26) (Figure 3B).

## 4. Discussion

In this paper, we investigated whether adult dogs and 14–16-month-old infants would show auditory–visual cross-modal matching when presented with the same dog and human picture accompanied with a dog’s bark and human speech. Overall, both dogs and infants showed evidence of intermodal matching. (i) Dogs looked first at the congruent picture that corresponded to the sound played back even if the vocaliser was a heterospecific, while their looking duration provided evidence of intermodal matching only for conspecifics. (ii) Similar to dogs, infants looked longer at the dog picture while hearing the bark; however, they did not adjust their first fixation to the voice congruent picture and, compared with dogs, they fixated more at the picture of the heterospecific when it was voice incongruent.

When the dog bark was emitted, dogs looked longer at the dog picture than at the human portrait, but they did not spend more time looking at the human portrait compared with the dog picture when the speech was played. Previous studies suggested that dogs prefer looking at the picture of a conspecific to looking at a human portrait, for example [29,39]. Preferential looking at conspecifics has been demonstrated in various species (e.g., chimpanzees [40], lemurs [41]) potentially because of the fact that conspecific faces might contain more information, and thus attract subjects’ attention to a greater extent. On this basis, we can assume that this general preference for pictures of conspecifics is responsible for the lack of intermodal matching upon hearing the Speech in dogs. However, the results of the analysis of dogs’ first look and the fact that they spent less time looking at the dog picture during the speech than during the bark indicate that they associated the human voice with the human portrait as well.

Interestingly, the pattern of infants’ looking duration was similar to that observed in dogs. Namely, effective picture–voice matching occurred only upon hearing the dog bark. At the same time, their looking preference toward the dog picture decreased marginally upon hearing human speech, which suggests cross-modal association between the human voice and human face. In line with this assumption, numerous previous studies showed that infants can associate appropriate human pictures and voices from an early age in various contexts [1,6,42,43]; therefore, it is improbable that 14–16-month-old infants had difficulty in matching the visual and auditory stimuli of the conspecific in the present study. Rather, their looking pattern was potentially similarly guided by a preference for dogs. Note that there may be different reasons for infants’ relative preference for dog versus human images. (i) It may be because of the so-called ‘novelty effect’ (i.e., when the novel stimulus captures more attention than a familiar one [44]), (ii) it can also refer to a more specific pre-experimental preference toward looking at dogs in infants at this particular age [45], or (iii) it might be because of the methodological features of the present study (i.e., experimental stimuli and design). Below, we consider these possibilities in more detail.

(i) We hypothesised that from the infants’ perspective, the dog bark and portrait were more novel than an unfamiliar human picture and voice, therefore, a preference for novelty would increase infants’ attention toward the picture of the non-conspecific. Preference for novel stimuli is a general phenomenon in human infants and serves as a basis for the widely-used habituation paradigm [44,46,47]. Numerous studies on unimodal visual perception showed that infants’ preferential looking behaviour varies with their age [48] as well as with the given task (for a review, see [49]). At the same time, little to no evidence is available concerning whether and how intersensory matching ability changes over time. Nonetheless, if the novelty preference account is correct, then our results do not contradict the idea that cross-species intersensory perception does not decline with infants’ age [9], but merely suggests that it may not be manifested at all times because of a novelty preference.

(ii) Another possible explanation of infants’ looking behaviour in the present study is the so-called ‘pre-experimental’ or ‘spontaneous’ category preference (i.e., when the subject has a pre-experimental preference toward certain types of stimuli; for a review, see [49]). It has been shown that 3–4-month-old infants show an a prior (i.e., pre-experimental) preference for dog faces over cat faces [45], and 3–4-month-old, but not 6–7-month-old infants prefer to look at cats over horses and tigers [47]. On the basis of these results, it is possible that 14–16-month-old infants in the present experiment have an attentional preference toward the dog picture, which resulted in elevated looking durations toward the non-conspecific.

(iii) The third possibility concerns the methodological specifications of the present study. Using only one dog and human picture and the corresponding vocalisations is a limitation of our study. Thus, it is also possible that this particular set of stimuli elicited this pattern of looking behaviour in infants.

Further investigations are needed to clarify which hypothesis explains infants’ looking behaviour in the present study. Moreover, using pictures and vocalisations of various people and dogs could provide further support for the conclusion that the association observed in the current study is general enough to cover “species recognition”, and would also help to expand the scope of investigations about cross-modal species recognition in infants and dogs.

It has been raised that dogs and humans went through a co-evolutionary process during which the behaviour of both species has changed significantly [50,51,52,53]. For instance, dogs vocalise more and in a wider range of circumstances than wolves [54] and the acoustic parameters of their vocalisations provide information for humans about the inner state of the dog [36]. It has been recently shown that the dog and human brains have similar voice-sensitive regions. Furthermore, acoustical cues related to emotional valence of both con- and non-conspecific vocalisations are processed similarly in the dog and human auditory cortex [55].

Regarding cross-species intersensory perception, it has been raised that infants’ ability to match non-conspecific (i.e., monkey) vocalisations and faces declines with age [8,27]. It has been also suggested that this decline after 10 months of age is experience-based and is due to perceptual narrowing toward more relevant species-specific features [27]. Contrary to these findings, but in line with the results of a recent study [9], 14–16-month-old infants in the present experiment showed clear auditory–visual matching of a dog picture and vocalisation. If we assume that cross-species intermodal matching ability narrows with age to relevant faces and vocalisations, these results might indicate that dog faces and barks convey relevant information for humans. Adult humans are able to classify dog barks recorded in different situations, irrespective of previous experience with dogs [36], which further suggests that this ability does not decline for canine vocalisations and raises the possibility that convergent evolution between humans and dogs may have led to the acquisition of a more permanent cross-species intermodal matching ability in humans.

Taken together, our results showed effective cross-modal matching in dogs and infants and provided further evidence that acoustic and visual information of dogs and humans is informative for both species and might serve as a basis to identify the corresponding con- and heterospecific signaller.

## 5. Conclusions

In the present study, we used a non-invasive eye-tracker method in order to investigate and directly compare dogs’ and infants’ auditory-visual matching abilities when presented with pictures and vocalisations of a dog and a female human. Interestingly, only dogs provided evidence of both con- and heterospecific intermodal matching, while infants’ showed clear preference toward the dog versus the human picture, especially when it was voice congruent. We assumed that looking preference of infants was rather due to stimulus novelty, pre-experimental preference, or methodological features of the current study than to their inability to match corresponding faces and voices of the conspecific.

## Supplementary Materials

The following are available online at http://www.mdpi.com/2076-2615/9/1/17/s1: S1 Data. Raw looking data of dogs and infants (XLSX).
